# Distribution of Alox15 in the Rat Brain and Its Role in Prefrontal Cortical Resolvin D1 Formation and Spatial Working Memory

**DOI:** 10.1007/s12035-017-0413-x

**Published:** 2017-02-08

**Authors:** Suku-Maran Shalini, Christabel Fung-Yih Ho, Yee-Kong Ng, Jie-Xin Tong, Eng-Shi Ong, Deron R. Herr, Gavin S. Dawe, Wei-Yi Ong

**Affiliations:** 1 Department of Anatomy, National University of Singapore, Singapore, 119260 Singapore; 2 Neurobiology and Ageing Research Programme, National University of Singapore, Singapore, 119260 Singapore; 3 Department of Science, Singapore University of Technology and Design, Singapore, 487372 Singapore; 4 Department of Pharmacology, National University of Singapore, Singapore, 119260 Singapore

**Keywords:** 15 Lipoxygenase 1, Learning and memory, Long-term potentiation, Spatial working memory, Synaptic plasticity, DHA, Resolvin D1

## Abstract

Docosahexaenoic acid (DHA) is enriched in membrane phospholipids of the central nervous system (CNS) and has a role in aging and neuropsychiatric disorders. DHA is metabolized by the enzyme Alox15 to 17S-hydroxy-DHA, which is then converted to 7S-hydroperoxy,17S-hydroxy-DHA by a 5-lipoxygenase, and thence via epoxy intermediates to the anti-inflammatory molecule, resolvin D1 (RvD1 or 7S,8R,17S-trihydroxy-docosa-Z,9E,11E,13Z,15E,19Z-hexaenoic acid). In this study, we investigated the distribution and function of Alox15 in the CNS. RT-PCR of the CNS showed that the prefrontal cortex exhibits the highest Alox15 mRNA expression level, followed by the parietal association cortex and secondary auditory cortex, olfactory bulb, motor and somatosensory cortices, and the hippocampus. Western blot analysis was consistent with RT-PCR data, in that the prefrontal cortex, cerebral cortex, hippocampus, and olfactory bulb had high Alox15 protein expression. Immunohistochemistry showed moderate staining in the olfactory bulb, cerebral cortex, septum, striatum, cerebellar cortex, cochlear nuclei, spinal trigeminal nucleus, and dorsal horn of the spinal cord. Immuno-electron microscopy showed localization of Alox15 in dendrites, in the prefrontal cortex. Liquid chromatography mass spectrometry analysis showed significant decrease in resolvin D1 levels in the prefrontal cortex after inhibition or antisense knockdown of Alox15. Alox15 inhibition or antisense knockdown in the prefrontal cortex also blocked long-term potentiation of the hippocampo-prefrontal cortex pathway and increased errors in alternation, in the T-maze test. They indicate that Alox15 processing of DHA contributes to production of resolvin D1 and LTP at hippocampo-prefrontal cortical synapses and associated spatial working memory performance. Together, results provide evidence for a key role of anti-inflammatory molecules generated by Alox15 and DHA, such as resolvin D1, in memory. They suggest that neuroinflammatory brain disorders and chronic neurodegeneration may ‘drain’ anti-inflammatory molecules that are necessary for normal neuronal signaling, and compromise cognition.

## Introduction

Lipoxygenases (EC 1.13.11) are a superfamily of oxidoreductive enzymes that contain a nonheme iron and catalyze the incorporation of two atoms of oxygen into a single donor, specifically polyunsaturated fatty acids (PUFA) containing a cis,cis-1,4- pentadiene structure [1, 2]. The dioxygenation of PUFAs such as docosahexaenoic acid (DHA) and arachidonic acid (AA) by lipoxygenases generates fatty acid hydroperoxy products [1, 3]. Of the lipoxygenases, 15-lipoxygenase catalyzes the direct dioxygenation of phospholipids and cholesterol esters of biological membranes and plasma lipoproteins [4]. There are two isoforms of the 15-lipoxygenase enzyme, the type 1 (Alox15), also known in rodents as leukocyte-type and in humans as reticulocyte-type, and type 2 (Alox15B) [5], both of which regulate the production of fatty acid hydroperoxides [6, 7]. Human reticulocyte-type Alox15, also known as arachidonate 15-lipoxygenase (Alox15), is a 75 kDa enzyme made up of a single chain of amino acids. The Alox15 gene is found on human chromosome 17. It has 10 tandem repeats of a motif rich in pyrimidine in its 3′-untranslated region, which regulate enzyme expression inhibiting Alox15 translation through association with the regulatory proteins, heterogeneous ribonucleoprotein K, and heterogeneous ribonucleoprotein E1 [8].

Alox15 metabolizes DHA to 17S-hydroxy-DHA, which is then converted to 7S-hydroperoxy,17S-hydroxy-DHA by a 5-lipoxygenase, and thence via epoxy intermediates to resolvin D1 (RvD1 or 7S,8R,17S-trihydroxy-docosa-Z,9E,11E,13Z,15E,19Z-hexaenoic acid) and resolvin D2 (RvD2 or 7S,16R,17S trihydroxy-docosa-4Z,8E,10Z,12E,14E,19Z-hexaenoic acid) [9]. The Alox15 product 17S-hydroxy-DHA can also be converted to a 16(17)-epoxide and then to the 10,17-dihydroxy docosatriene termed neuroprotectin D1 (NPD1) [9]. These lipid mediators are involved in the removal of inflammatory cells and restoration of tissue integrity during resolution of inflammation [10]. They can modulate the effects of proinflammatory eicosanoids derived from AA [11], reduce leukocyte trafficking, and downregulate cytokine expression [11]. Alox15 also plays a role in supraspinal antinociception originating in the prefrontal cortex [12], but thus far, little is known about the distribution and function of the enzyme in the CNS.

The hippocampus and prefrontal cortex function together jointly as a memory system enabling working memory and consolidation of contextual information [13]. DHA and its docosanoids are beneficial for these cognitive functions [14]. DHA, which is a substrate of Alox15, is enriched in membrane phospholipids of the central nervous system (CNS) [15], and disturbances in its metabolism could play a role in aging and neuropsychiatric disorders [15–17]. DHA is released from membrane phospholipids by calcium-independent phospholipase A_2_ (iPLA_2_) [18–21]. Decreased iPLA_2_ activity has been detected in the prefrontal cortex of patients with Alzheimer’s disease (AD) [22]. iPLA_2_ plays a role in synaptic plasticity, and inhibition or antisense oligonucleotide knockdown of iPLA_2_ prevents induction of hippocampo-prefrontal cortex long-term potentiation (LTP) [23]. Selective inhibitors of iPLA_2_ prevent induction of LTP in hippocampal slices [24]. DHA rescues the impairment caused by an iPLA_2_ inhibitor [25, 26] while its supplementation improves learning and memory in patients with age-related cognitive decline [27] and protects from amyloid and dendritic pathology in AD model mice [28–30].

We hypothesize that DHA and its metabolites that are produced by the action of iPLA_2_ are important for hippocampo-prefrontal cortex synaptic plasticity and prefrontal cortex-dependent working memory. The present study was carried out to elucidate the relative expression of Alox15 across brain regions and its possible role in prefrontal cortical function.

## Methods and Materials

### Animals

Adult male Wistar rats (250–300 g) were purchased from InVivos, Singapore, and housed in temperature controlled (23 ± 1 °C), individually ventilated cages on a 12-h light-dark cycle (7AM–7PM) with access to food and water. Rats were acclimatized for 4 days before the start of experiments. All procedures were in accordance with the Principles of Laboratory Animal Care and approved by the Institutional Animal Care and Use Committee of the National University of Singapore.

### Chemicals

The specific Alox15 inhibitor, PD146176, was purchased from Cayman Chemicals (Ann Arbor, Michigan, USA) and was diluted in the vehicle dimethyl sulfoxide (DMSO) (Sigma, St. Louis, USA).

### Antisense Oligonucleotide

The antisense oligonucleotide used was a 16-base oligonucleotide (5′-CACATGGTGATGAAGT-3′). This has been shown to effectively knock down Alox15 expression in the mouse brain [12], but is also suitable for the rat brain. Scrambled sense oligonucleotide was used as a control (5′-CACGTCTATACACCAC-3′). Both antisense and sense oligonucleotides contained phosphorothioate linkages to prevent nuclease degradation (IBA, Germany). Solutions of 200 μM were prepared by dissolving lyophilized material in nuclease-free water.

### Stereotaxic Injection

Rats were anesthetized with the inhalational anesthetic isoflurane (Sigma-Aldrich, St Louis, USA)—up to 5% for induction, 1–3% for maintenance using a precision vaporizer and mounted on a stereotaxic frame (Stoelting, Wood Dale, USA). A midline incision was made on the scalp and small craniotomies performed over the injection sites, 4.0 mm anterior and 1.5 mm lateral to the bregma on both sides, and 2.0 mm from the surface of the cortex. These coordinates correspond to the prefrontal cortex and were determined using the rat brain atlas of Paxinos and Watson 1998 [31]. Five microliters of either vehicle control (DMSO) or Alox15 inhibitor in DMSO (40 mM) and 2 μl of antisense oligonucleotide or scrambled sense oligonucleotide were bilaterally injected into the cortex, at a rate of 5 min per injection. Injections were carried out in a blinded manner to reduce experimenter bias.

### Real-Time Reverse Transcriptase Polymerase Chain Reaction

Six adult male Wistar rats were used per experiment for this portion of the study. Rats were deeply anesthetized with a ketamine/xylazine cocktail and sacrificed by decapitation. The ketamine/xylazine mixture used was prepared in saline [32] (7.5 ml ketamine (75 mg/kg), 5 ml xylazine (10 mg/kg), in 7.5-ml 0.9% sodium chloride solution). Ketamine used was obtained from Parnell Manufacturing Pte Ltd., Alexandria, New South Wales, Australia, while xylazine used was obtained from Ilium Xylazil, Troy Laboratories Pty Ltd., Glendenning, New South Wales, Australia. Various parts of the rat brain including olfactory bulb, prefrontal cortex, cortex 1, cortex 2, striatum, thalamus/hypothalamus, hippocampus, cerebellum, and brainstem were dissected out, immersed in RNAlater® solution (Ambion, TX, USA), and snap frozen in liquid nitrogen. Anatomically, cortex 1 contains the primary and secondary motor cortex and the primary somatosensory cortex, whereas cortex 2 includes the parietal association cortex and secondary auditory cortex.

Total RNA was extracted using Trizol reagent (Invitrogen, CA, USA) according to (manufacturer’s) protocol. RNeasy® Mini Kit (Qiagen, Inc., CA, USA) was used to purify the RNA. Samples were reverse transcribed using HighCapacity cDNA Reverse Transcription Kits (Applied Biosystems, CA, USA). Reaction conditions were 25 °C for 10 min, 37 °C for 120 min, and 85 °C for 5 s. Real-time PCR amplification was performed using a 7500 Real-time PCR system (Applied Biosystems) with TaqMan® Universal PCR Master Mix (Applied Biosystems), Alox5 (Rn00563172_m1), Alox12 (Rn01461082_m1), Alox15 (Rn00696151_m1), and β-actin probes (#4352340E) (Applied Biosystems, CA, USA) according to the manufacturer’s instructions. The PCR conditions were as follows: incubation at 50 °C for 2 min and 95 °C for 10 min followed by 40 cycles of 95 °C for 15 s and 60 °C for 1 min. All reactions were carried out in triplicate. The threshold cycle (C_T_) was measured as the number of cycles in which the reporter fluorescence emission exceeds the preset threshold level. Amplified transcripts were quantified using the comparative C_T_ method [33], with the formula for relative fold change =2^−∆∆CT^. The mean and standard error were then calculated.

### Western Blot

Six adult male Wistar rats were used for the first portion of this study. Rats were deeply anesthetized with a ketamine/xylazine cocktail and sacrificed by decapitation. Various parts of the rat brain including olfactory bulb, prefrontal cortex, striatum, thalamus/hypothalamus, hippocampus, cerebellum, brainstem, and spinal cord were dissected out and snap frozen in liquid nitrogen.

Four adult male Wistar rats were used per group (scrambled sense and Alox15 antisense) for the second portion of this study. Rats that had been stereotaxically injected with scrambled sense or antisense oligonucleotides were deeply anesthetized with the ketamine/xylazine cocktail and sacrificed by decapitation 4 days after stereotaxic injection. The prefrontal cortices of these rats were dissected out and snap frozen in liquid nitrogen.

Tissues were homogenized using a Tissue Tearor® (Biospec, ITS Science and Medical, Singapore) in ice-cold buffer (M-Per mammalian protein extraction kit, 1 mM EDTA and 0.25 mM DTT). Homogenates were centrifuged at 13,000 rpm for 20 min, and the supernatant collected. Protein concentration in the supernatant was determined using a Bio-Rad protein assay kit (Bio-Rad Laboratories, Hercules, USA). Proteins were resolved in 15% sodium dodecyl sulfate polyacrylamide gel (SDS-PAGE) under reducing conditions and subsequently electrotransferred to a PVDF membrane (Amersham Pharmacia Biotech, Little Chalfont, UK). The molecular weights of the proteins were determined using a Bio-Rad Prestained Protein Ladder (Bio-Rad Laboratories). Non-specific binding sites on the PVDF membrane were blocked by incubation with 5% non-fat milk in Tris-buffered saline-Tween (TBST) for 1 h. The PVDF membrane was then incubated overnight with an affinity-purified mouse monoclonal antibody to Alox15 (Abcam, Cambridge, UK, diluted 1:2000) in TBST with 5% non-fat milk. The membrane was then washed in TBST and incubated with horseradish peroxidase conjugated horse anti-mouse IgG (ThermoFisher Scientific, Waltham, USA, diluted 1: 2000) for 1 h at room temperature. Immunoreactivity was visualized using an enhanced chemiluminescence kit (Pierce, Rockford, USA) according to the manufacturer’s instructions. Band intensities were quantified by densitometric analysis.

### Immunohistochemistry

Four adult male Wistar rats were used in this portion of the study. Rats were deeply anesthetized and perfused through the left cardiac ventricle with a solution of 4% paraformaldehyde and 0.1% glutaraldehyde in 0.1 M phosphate buffer (pH 7.4). The brains were removed and sectioned coronally at 100 μm using a vibrating microtome. Sections were washed 30 times for 5 min each with phosphate-buffered saline (PBS) and incubated overnight with an affinity-purified mouse monoclonal antibody to Alox15 (Abcam, Cambridge, UK), diluted 1:50 in PBS. Sections were incubated for 1 h in a 1:100 dilution of biotinylated horse anti-mouse IgG (Vector, Burlingame, CA), reacted for 1 h with avidin-biotinylated horseradish peroxidase complex, and visualized by treatment for 22 min in 0.05% 3,3-diaminobenzidine tetrahydrochloride solution in Tris buffer containing 0.05% hydrogen peroxide. Some sections were mounted on glass slides and counterstained with methyl green and visualized with a light microscope. The remaining sections were processed for electron microscopy.

### Electron Microscopy

Electron microscopy was carried out by subdissecting immunostained sections of the prefrontal cortex into smaller rectangular portions (1.0 × 1.5 mm). Samples were osmicated for 1 h in osmium tetroxide (OsO_4_), washed in distilled water for 20 min, then dehydrated in an ascending series of ethanol and acetone as follows: 25% ethanol, 3 min; 50% ethanol, 5 min; 75% ethanol, 5 min; 95% ethanol, 5 min; 100% ethanol, 5 min; 100% acetone, 5 min × 2, then embedded in Araldite. Thin sections were obtained from the first 5 μm of the sections, mounted on copper grids coated with Formvar, and stained with lead citrate. They were viewed using a JEOL 1010EX electron microscope.

### Liquid Chromatography Mass Spectrometry

Six adult male Wistar rats were used per group (vehicle control and Alox15 inhibitor) in the first portion of this study. Rats were stereotaxically injected with 5 μl of either vehicle control (DMSO) or Alox15 inhibitor in DMSO (40 mM) bilaterally at a rate of 5 min per injection as described above. After a 1-day time point, rats were deeply anesthetized with the ketamine/xylazine cocktail and sacrificed by decapitation. The prefrontal cortex of these rats was removed and snap frozen in liquid nitrogen.

Six adult male Wistar rats were used per group (saline, Alox15 scrambled sense, and Alox15 antisense) in the second portion of this study. Rats were stereotaxically injected with either scrambled sense oligonucleotide, Alox15 antisense oligonucleotide, or saline bilaterally at a rate of 5 min per injection as described above. The prefrontal cortex was removed and snap frozen in liquid nitrogen.

Tissues were homogenized in 750 μl of Folch solution (2:1 *v*/*v* chloroform/methanol) using a Tissue Tearor™ (Biospec, USA). Samples were then sonicated for 30 min at 4 °C. A total of 200 μl of 0.88% KCl was added to each sample. Samples were vortexed for 1 min, then centrifuged at 9000*g* for 2 min, and the organic portions collected and vacuum-dried (Thermo Savant SpeedVac, USA). Lipids were then resuspended in 200 μl of 100% acetonitrile and transferred to an amber glass vial for LC/MS analysis.

LC/MS assay was carried out using a Shimadzu LC system equipped with a binary gradient pump, auto-sampler, column oven, and diode array detector, coupled with a Shimadzu LCMS 8060 triple quadrupole mass spectrometer (Kyoto, Japan). Gradient elution involved a mobile phase consisting of (A) 0.1% formic acid in water and (B) 0.1% formic acid in acetonitrile. The initial condition was set at 5% of (B), gradient up to 100% in 10 min and returning to initial condition for 5 min. Oven temperature was set at 40 °C and flow rate was set at 250 μl/min. For all experiments, 2 μl of samples was injected. The column used for the separation was a reversed-phase Zorbax SB18, 50 × 2.0 mm, 3.5 μm (Agilent Technologies, USA). The ESI/MS was acquired in the positive and negative ion mode. Product ions of m/z range from 100 to 800 were collected. The drying gas and nebulizer nitrogen gas flow rates were 10 l/min and 1.5 l/min respectively. The DL temperature was °C and BH temperature was 400 °C. The LC/MS data were peak-detected and noise reduced, such that only true analytical peaks were further processed. A list of the intensities of the peaks detected was then generated manually and tabulated into Microsoft Excel for each sample run, using the retention tine (RT) and m/z data pairs as the identifier for each peak. The ion intensities for each peak detected were normalized within each sample, to the sum of the peak intensities in that sample. Differences in the amount of lipid species present were normalized to total amount of lipids in each sample and analyzed using one-way ANOVA or two-tailed unpaired Student’s *t* test. *P* < 0.05 was considered significant.

### In Vivo Electrophysiology

Six adult male Wistar rats were used per treatment group (vehicle control, Alox15 inhibitor, scrambled sense oligonucleotide, and Alox15 antisense oligonucleotide) in this portion of the study. Rats underwent in vivo electrophysiology testing 1 day after intracortical injection (for inhibitor studies) and 4 days after intracortical injection (for antisense studies). The in vivo electrophysiology procedure was conducted as described previously [34]. Rats were anesthetized with urethane (Sigma-Aldrich) and mounted on a stereotaxic frame (Stoelting). Urethane was freshly prepared in sterile isotonic saline (0.9% sodium chloride solution) before use, at a concentration of 1 g/kg body weight. Body temperature was maintained at 37 °C by a homeothermic blanket. A midline incision was made on the scalp and small craniotomies performed using a burr over the sites of insertion of the electrodes. A bipolar nichrome wire stimulating electrode was placed in the CA1/subicular region of the temporal hippocampus (6.3 mm posterior and 5.5 mm lateral to the bregma). A monopolar stainless steel recording electrode (SNE-300, David Kopf Instruments, Tujunga, USA) with a recording tip of diameter 100 μm and length 250 μm was placed in the prelimbic area of the prefrontal cortex (3.3 mm anterior and 0.9 mm lateral to the bregma). Coordinates were determined by reference to the atlas of Paxinos and Watson 1998 [31].

Stimulation of the CA1/subicular region of the hippocampus resulted in characteristic monosynaptic negative-going field potential recorded from the prefrontal cortex, with a latency of 18–24 ms. The depths of the stimulating and recording electrodes (5.8–7.2 mm and 3.2–4.7 mm from the skull surface, respectively) were adjusted to maximize the amplitude of negative-going peak of the evoked response. During the initial localization of the response, stimulation at varying intensities (between 200 and 350 μA) was delivered once every 15 s. Once an appropriate response was established, a period of 10 min was given to allow for response stabilization. The stimulus intensity required to evoke a response that was 70% of the maximal response was determined via rendering of an input/output curve (IOC) and used during the protocol. The stimulation protocol used for the experiment is as follows: baseline recording was performed once every 30 s for 30 min at the stimulus intensity determined by the IOC. High-frequency stimulation (HFS) was then delivered (50 pulses at 250 Hz, 4 ms interval between pulses). This sequence was repeated 10 times. After HFS, baseline stimulation was resumed, and recording was continued for at least 90 min. Five-minute averages of the amplitude were calculated for further analysis, and the average field excitatory post-synaptic potentials (fEPSP) expressed as mean percentage ± SEM normalized to baseline for each experiment. Differences between fEPSP (%) recorded for each treatment group were analyzed using repeated measures two-way ANOVA. *P* < 0.05 was considered significant.

### Rewarded Alternation in a T-Maze

Six adult male Wistar rats were used per group (vehicle control, Alox15 inhibitor, scrambled sense oligonucleotide, and Alox15 antisense oligonucleotide) in this portion of the study. The T-maze rewarded alternation testing procedure was conducted as previously described [35]. Rats were habituated for 5 days and trained for a further 5 days to alternate in the maze in order to obtain a food reward. On day 11, rats underwent intracortical injection of vehicle control, Alox15 inhibitor, scrambled sense oligonucleotide, or Alox15 antisense oligonucleotide in a blinded manner. They were subsequently tested on the T-maze. Differences in the number of correct options (entry into previously unentered arm) were analyzed using the two-tailed unpaired Student’s *t* test. *P* < 0.05 was considered significant.

## Results

### Real-Time Reverse Transcriptase Polymerase Chain Reaction

**Fig. 1:**
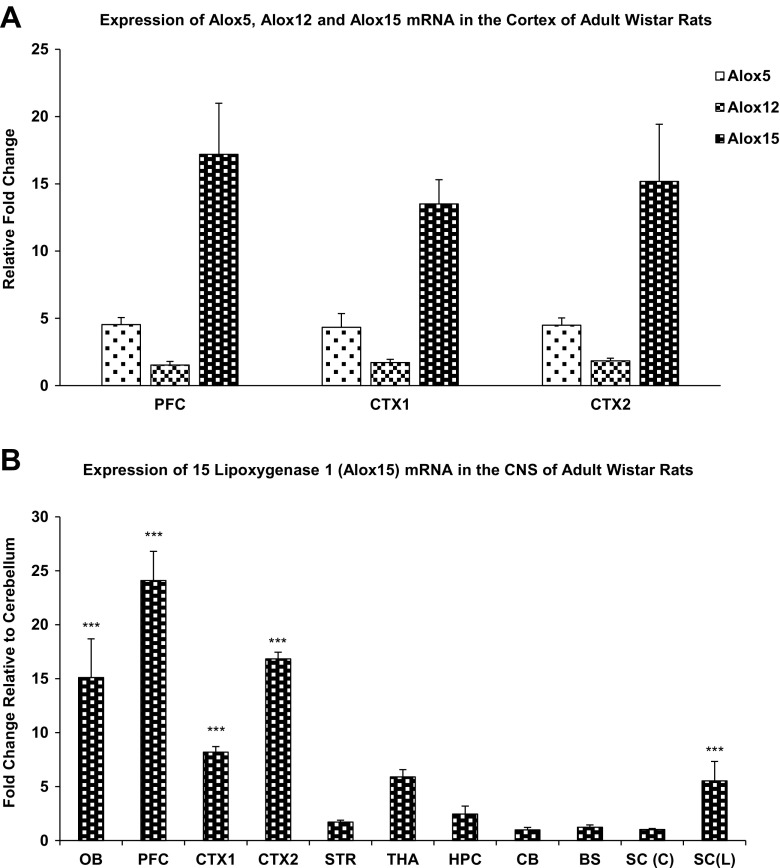
**a** Differential expression of Alox5, Alox12, and Alox15 mRNA in the cortex of adult Wistar rats. Anatomically, cortex 1 contains the primary and secondary motor cortex and the primary somatosensory cortex, whereas cortex 2 includes the parietal association cortex and secondary auditory cortex. **b** Differential expression of Alox15 mRNA in the CNS of adult Wistar rats in the olfactory bulb, prefrontal cortex, cortex, striatum, hippocampus, thalamus, cerebellum, brainstem, and spinal cord. *Asterisks* indicate significant differences relative to cerebellum at *P < 0.05, **P < 0.01, ***P < 0.001, one-way ANOVA with Bonferroni post hoc test. Abbreviations: *OB* olfactory bulb, *PFC* prefrontal cortex, *CTX1* cortex 1, *CTX2* cortex 2, *STR* striatum, *HC* hippocampus, *CX* cerebral cortex, *SE* septum, *ST* striatum, *HC* hippocampus, *TH* thalamus, *CB* cerebellum, *BS* brainstem, *SC(C)* cervical region of spinal cord, *SC(L)* lumbar region of spinal cord

Real-time RT-PCR results showed that Alox15 is the highest-expressing isoform in the rat cortex, compared to Alox5 and Alox12. Alox15 mRNA expression was higher than those of Alox5 and Alox12 in the prefrontal cortex, the primary and secondary motor cortex, the primary somatosensory cortex, the parietal association cortex, and secondary auditory cortex (Fig. 1a).

After normalization to the endogenous control, β-actin, the relative mRNA expression of Alox15 was determined in each brain region relative to the area with lowest expression, the cerebellum. Real-time RT-PCR results indicate that the prefrontal cortex exhibits the highest mRNA expression level with approximately 25-fold greater expression than the cerebellum, followed by the parietal association cortex and secondary auditory cortex with over 15-fold greater expression than the cerebellum, olfactory bulb with approximately 15-fold greater expression than the cerebellum, motor and somatosensory cortices, and the hippocampus. Higher Alox15 mRNA levels are expressed across the forebrain regions as compared to the hindbrain and the spinal cord (Fig. 1b).

### Western Blot Analysis of Alox15 Protein Expression in the Brain

**Fig. 2:**
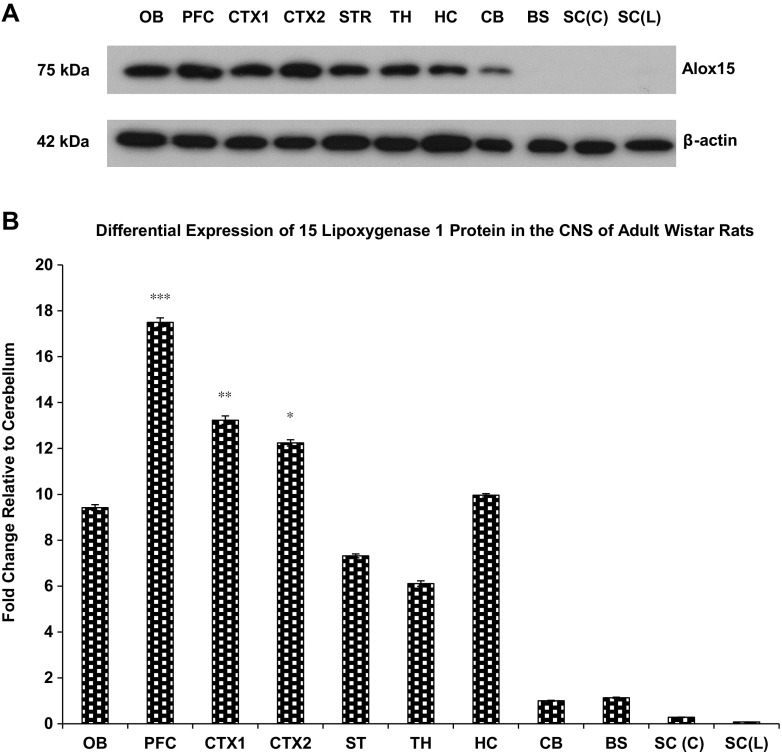
**a** Western blot of differential expression of Alox15 protein expression in the CNS of adult Wistar rats in the olfactory bulb, prefrontal cortex, cortex, striatum, hippocampus, thalamus, cerebellum, brainstem, and spinal cord. **b** Densitometric analyzes differential expression of Alox15 protein expression in the CNS of adult Wistar rats. *Asterisks* indicate significant differences relative to cerebellum at *P < 0.05, **P < 0.01, ***P < 0.001, one-way ANOVA with Bonferroni post hoc test. Abbreviations: *OB* olfactory bulb, *PFC* prefrontal cortex, CTX1 cortex 1, *CTX2* cortex 2, *ST* striatum, *HC* hippocampus, *CX* cerebral cortex, *SE* septum, *ST* striatum, *HC* hippocampus, *TH* thalamus, *CB* cerebellum, *BS* brainstem, *SC(C)* cervical region of spinal cord, *SC(L)* lumbar region of spinal cord

The Alox15 antibody detected a single 75 kDa band in the adult rat brain (Fig. 2a). The 75 kDa band size is consistent with the predicted 75 kDa Alox15 protein size [6], and to date, no significant glycosylation of the enzyme has been identified nor are there any indications of myristoylation or isoprenylation [36, 37]. Quantification of protein was determined by densitometric analysis of Alox15 bands normalized to that of β-actin.

To maintain consistency in comparison across real-time RT-PCR and Western blot, fold change levels were compared relative to the cerebellum. The prefrontal cortex was found to have the highest Alox15 protein expression with levels approximately 18-fold more than that of the cerebellum, followed by cortex 1 (13-fold), cortex 2 (12-fold), hippocampus (10-fold), and olfactory bulb (9-fold). The striatum and thalamus display relatively lower levels of protein expression while the hindbrain regions of cerebellum and brainstem and the spinal cord have low and insignificant levels of Alox15 expression. Results from the quantitative densitometric analysis of the Western blots are consistent with those of real-time RT-PCR. Overall, the prefrontal cortex expresses the highest Alox15 mRNA and protein expression levels relative to the cerebellum, followed by the cerebral cortical areas (Fig. 2b).

### Immunohistochemistry

**Fig. 3:**
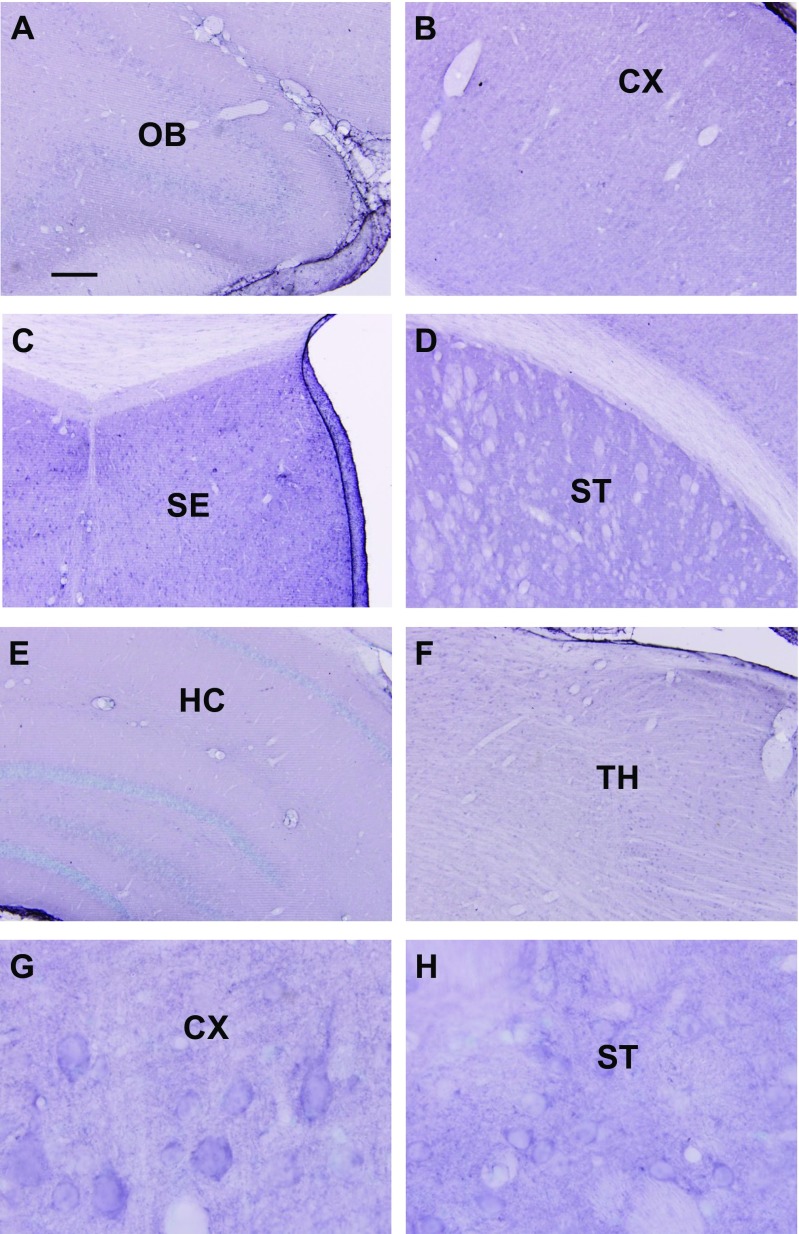
Immunohistochemical labeling of Alox5 in the forebrain. Moderately dense staining is observed in the olfactory bulb (**a**), cerebral cortex, including the prefrontal cortex (**b**), septum (**c**), striatum (**d**), while light staining is observed in the hippocampus (**e**), thalamus (**f**), and hypothalamus. Staining is mostly observed as punctuate profiles in the neuropil in these regions, and cell bodies were mostly unlabeled (**g**, **h**). Abbreviations: *OB* olfactory bulb, *CX* cerebral cortex, *SE* septum, *ST* striatum, *HC* hippocampus, *TH* thalamus. *Scale*: **a**–**f** = 200 μm. **g**, **h** = 20 μm

Moderately dense staining was observed in the olfactory bulb (Fig. 3a), cerebral cortex, including the prefrontal cortex (Fig. 3b), septum (Fig. 3c), striatum (Fig. 3d), while light staining was observed in the hippocampus (Fig. 3e), thalamus (Fig. 3f), and hypothalamus. Staining was mostly observed as punctuate profiles in the neuropil in these regions, and cell bodies were mostly unlabeled (Fig. 3g, h).

**Fig. 4:**
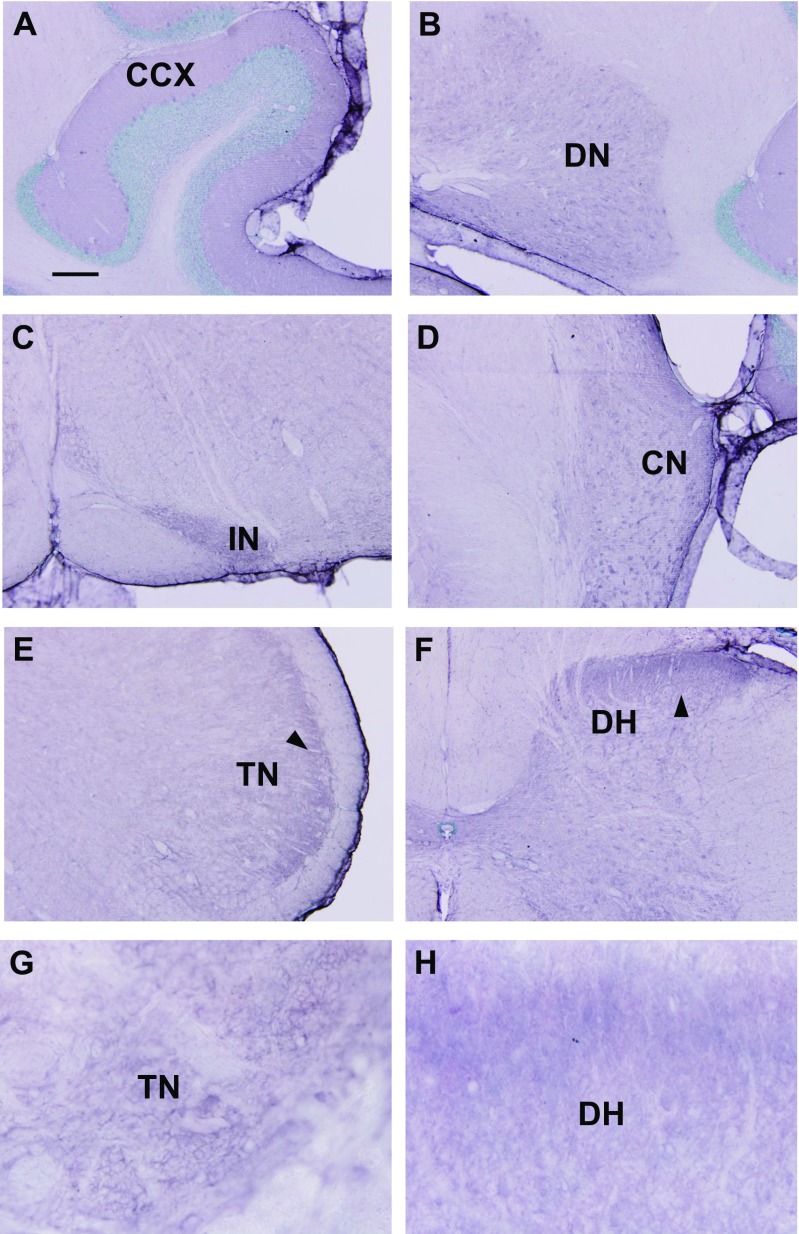
Immunohistochemical labeling of Alox5 in the hindbrain and spinal cord. The cerebellum including the cerebellar cortex (**a**) and deep cerebellar nuclei (**b**) were moderately labeled. Most parts of the brainstem are lightly labeled, except for the inferior olivary nucleus (**c**), dorsal and ventral cochlear nuclei (**d**), and superficial portion of the spinal trigeminal nucleus (**e**). The spinal cord is also lightly labeled, except the substantia gelatinosa, in the superficial part of the dorsal horn (**f**). Staining is mostly observed as punctuate profiles in the neuropil in these regions, and cell bodies were mostly unlabeled (**g**, **h**). Abbreviations: *CCX* cerebellar cortex, *DN* dentate nucleus, *IN* inferior olivary nucleus, *CN* cochlear nucleus, *TN* spinal trigeminal nucleus, *DH* dorsal horn of spinal cord (lumbar region). *Arrowheads* indicate immunoreaction product in superior portion of spinal trigeminal nucleus and dorsal horn. *Scale*: **a**–**f** = 200 μm. **g**, **h** = 20 μm

The cerebellum including the cerebellar cortex (Fig. 4a) and deep cerebellar nuclei (Fig. 4b) was moderately labeled. Most parts of the brainstem were lightly labeled, except for the inferior olivary nucleus (Fig. 4c), the dorsal and ventral cochlear nuclei (Fig. 4d), and the superficial portion of the spinal trigeminal nucleus (Fig. 4e). The spinal cord was also lightly labeled, except the substantia gelatinosa, in the superficial part of the dorsal horn (Fig. 4f). Staining was mostly observed as punctuate profiles in the neuropil in these regions, and cell bodies were mostly unlabeled (Fig. 4g, h).

### Electron Microscopy

**Fig. 5:**
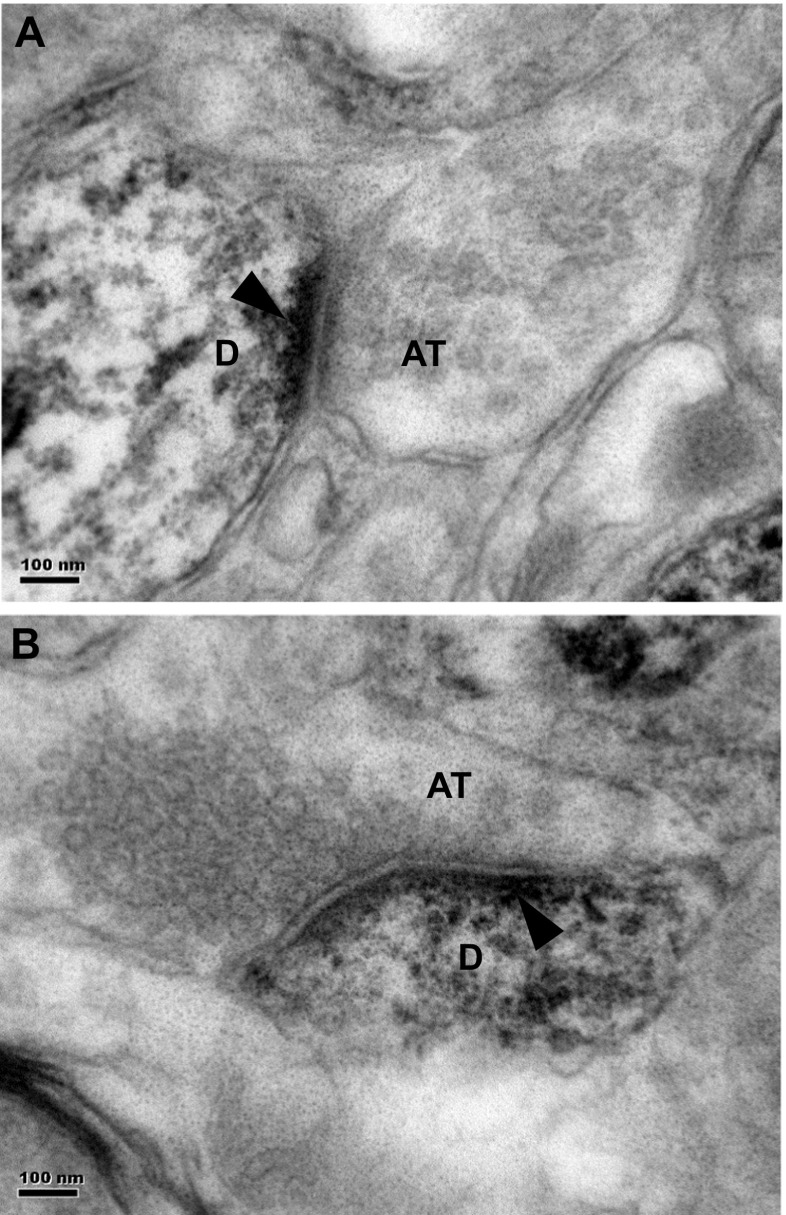
Electron microscopy of immunostained sections of the prefrontal cortex showed dense staining of the neuropil. Label was observed in dendrites or dendritic spines (**a**, **b**). *Arrowheads* indicate immunoreaction product. *Scale* = 100 nm. Abbreviations: *AT* axon terminal, *D* dendrite

Electron microscopy of immunostained sections of the prefrontal cortex showed dense staining of the neuropil. Label was observed in dendrites or dendritic spines (Fig. 5a, b).

### Western Blot Analysis to Confirm Alox15 Knockdown with Antisense Oligonucleotide

**Fig. 6:**
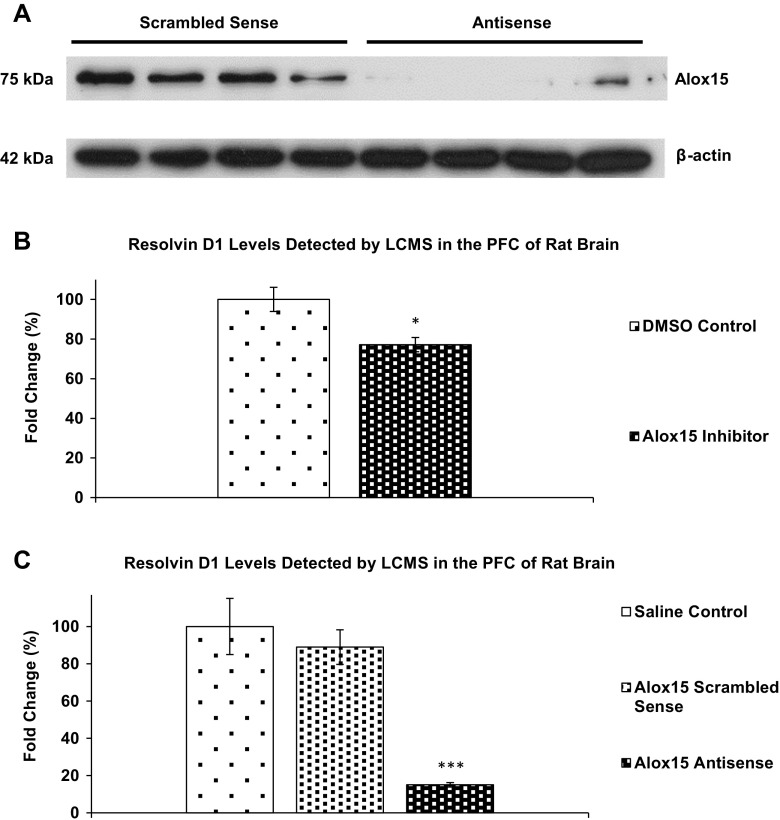
**a** Western blot of effect of Alox15 oligonucleotide treatment on Alox15 protein expression in the rat prefrontal cortex. **b** LCMS analysis of effect of Alox15 inhibitor treatment on resolvin D1 levels in the rat prefrontal cortex. **c** LCMS analysis of effect of Alox15 oligonucleotide treatment on resolvin D1 levels in the rat prefrontal cortex. *Asterisk* (*) indicate significant differences at *P* < 0.05, two-tailed Student’s *t* test. *Asterisks* (**) indicate significant differences at *P* < 0.01, one-way ANOVA with bonferroni post hoc test

The Alox15 antibody detected a single 75 kDa band in the adult rat brain (Fig. 6a), consistent with the predicted size of Alox15 protein. Analyses of normalized density of Alox15 bands to β-actin showed a significant 82% decrease (*P* < 0.05) in Alox15 protein levels in antisense-injected rats, indicating effective knockdown by Alox15 antisense oligonucleotide.

### Liquid Chromatography Mass Spectrometry

LCMS analysis shows a statistically significant decrease in resolvin D1 levels (*P* < 0.05) (Fig. 6b) after intracortical injection of Alox15 inhibitor in the prefrontal cortex of the rat brain.

LCMS analysis shows a statistically significant decrease in resolvin D1 levels (*P* < 0.01) (Fig. 6c) after intracortical injection of Alox15 antisense in the prefrontal cortex of the rat brain compared to saline and scrambled sense injected rats.

### In Vivo Electrophysiology

**Fig. 7:**
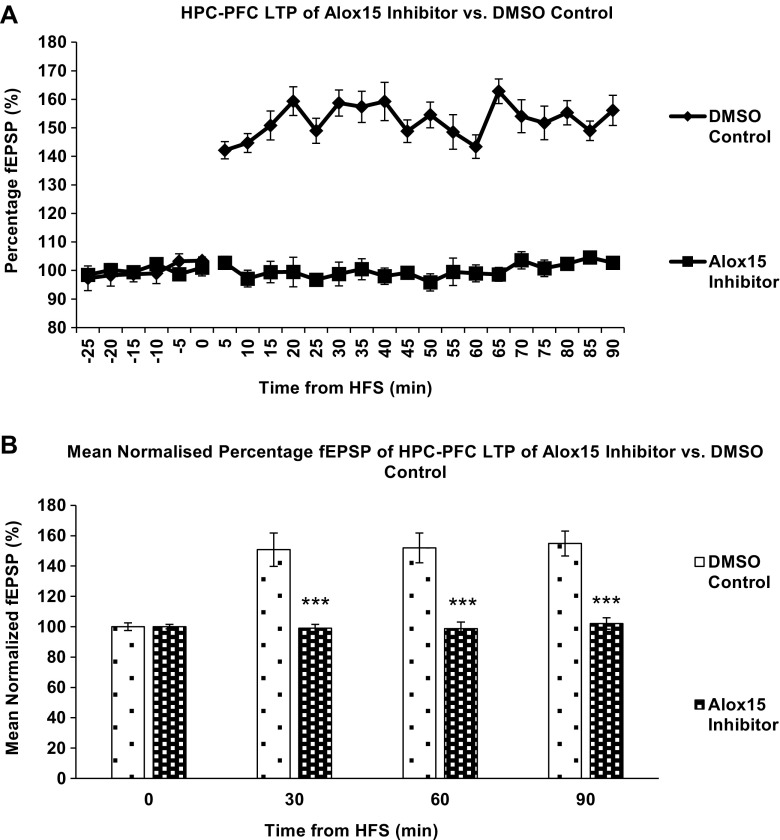
**a** Effects of hippocampal stimulation on fEPSP in prefrontal cortex. Data points represent mean fEPSP over the preceding 5 min. *Error bars* represent SEM. *n* = 6 for all treatment groups. Alox15 inhibitor treatment has a significant effect on fEPSP. *F*(1,10) = 34.759, *P* < 0.001. Repeated measures two-way ANOVA. **b** DMSO control vs Alox15 inhibitor. *Columns* represent mean normalized fEPSP over the preceding 30 min. *Asterisks* (***) indicate significant differences at *P* < 0.05, two-tailed Student’s *t* test

The effects of hippocampal stimulation on LTP induction in the hippocampo-prefrontal cortex pathway for rats treated with Alox15 inhibitor and DMSO vehicle control were evaluated by in vivo electrophysiology (Fig. 7a). Repeated measures two-way ANOVA indicated that HFS did induce LTP in control groups *F* (24,120) = 12.913, *P* < 0.001 and also indicated that the treatment (Alox15 inhibitor) exerted a significant effect on fEPSPs *F*(1,10) = 34.759, *P* < 0.001. Further, two-tailed Student’s *t* tests carried out to compare between the treatment groups showed no significant difference between fEPSPs of vehicle control and Alox15-injected rats before HFS, but confirmed a significant (*P* < 0.001) difference after HFS, sustained for 90 min post-HFS (Fig. 7b). This indicates that intracortical injection of Alox15 inhibitor prevented induction of LTP along the hippocampo-prefrontal cortex pathway.

**Fig. 8:**
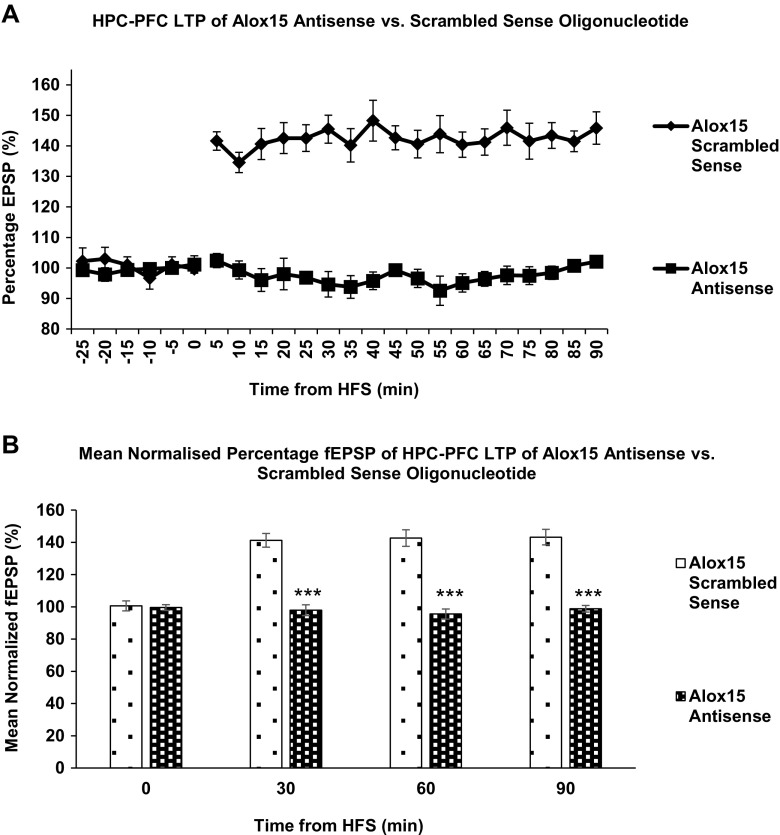
**a** Effects of hippocampal stimulation on fEPSP in prefrontal cortex. Data points represent mean fEPSP over the preceding 5 min. *Error bars* represent SEM. *n* = 6 for all treatment groups. Alox15 oligonucleotide treatment has a significant effect on fEPSP. *F* (1,10) = 156.178, *P* < 0.001. Repeated measures two-way ANOVA. **b** Scrambled sense control vs Alox15 antisense oligonucleotide. *Columns* represent mean normalized fEPSP over the preceding 30 min. *Asterisks* (***) indicate significant differences at *P* < 0.05, two-tailed Student’s *t* test

These results were confirmed with intracortical injection of Alox15 antisense oligonucleotides and scrambled sense control (Fig. 8a.) Repeated measures two-way ANOVA indicated that HFS did induce LTP in control groups *F* (24,120) = 26.315, *P* < 0.001 and also indicated that the treatment (Alox15 antisense) exerted a significant effect on fEPSP *F*(1,10) = 156.178, *P* < 0.001. Further, two-tailed Student’s *t* tests carried out to compare between the treatment groups showed no significant difference between fEPSPs of scramble sense and Alox15 antisense rats before HFS, but confirmed a significant (*P* < 0.001) difference after HFS, sustained for 90 min post-HFS (Fig. 8b). This confirms that genetic knockdown of Alox15 prevented induction of LTP along the hippocampo-prefrontal cortex pathway.

### Rewarded Alternation in T-Maze

**Fig. 9:**
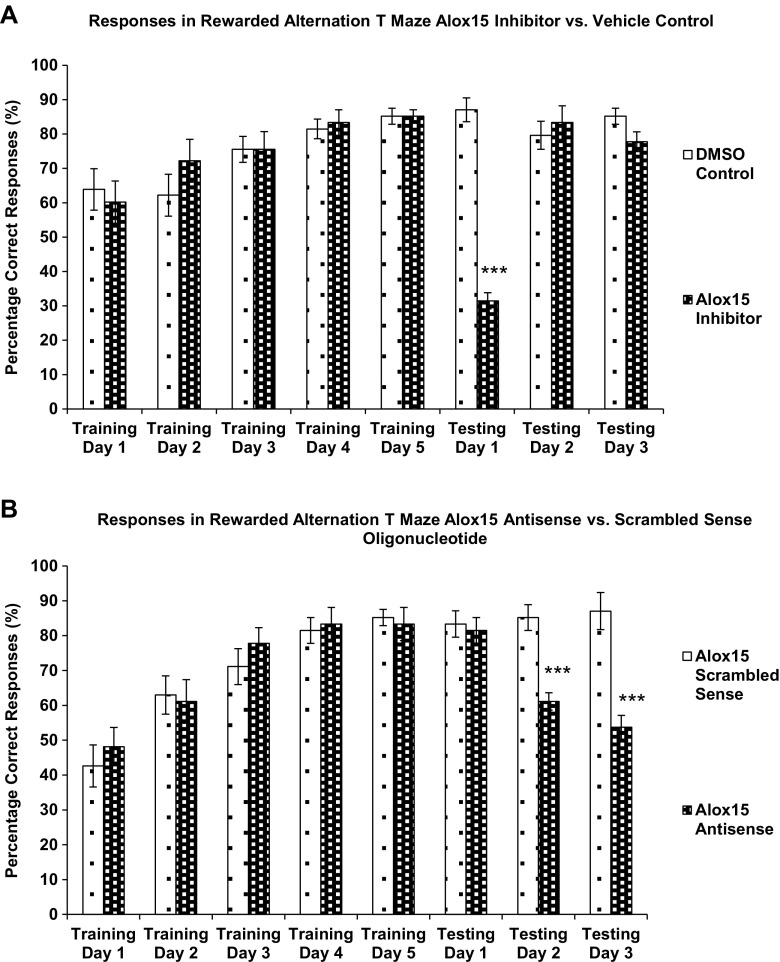
**a** Percentage correct responses made during T-maze procedure, DMSO control vs. Alox15 inhibitor. **b** Percentage correct responses made during T-maze procedure, Alox15 antisense oligonucleotide vs scrambled sense control. *Asterisks* (***) indicate significant differences at *P* < 0.05, two-tailed Student’s *t* test

Performance of rats in the rewarded alternation task in the T-maze was measured by the total number of correct responses (entry into previously unentered arm) made by a rat during the 5 training days and 3 testing days. Rats in the Alox15 and vehicle control groups performed equally well on training days 1–5. On testing day 1 (1 day after intracortical injection), rats injected with Alox15 inhibitor made significantly (*P* < 0.001) more errors than vehicle-injected controls. On testing days 2 and 3, performance of inhibitor-injected rats recovered to the same level as vehicle-injected controls (Fig. 9a).

Similarly, rats in the Alox15 antisense and scrambled sense control groups performed equally well on training days 1–5, while rats injected with Alox15 antisense oligonucleotide made significantly (*P* < 0.001) more errors than scrambled sense injected controls on testing days 2 and 3, which corresponds with the time taken for the antisense oligonucleotide to knock down protein expression (Fig. 9b). This confirms that the Alox15 enzyme in the prefrontal cortex is essential for spatial working memory.

## Discussion

The expression levels of reticulocyte-type Alox15 across the CNS were studied via real-time RT-PCR and Western blot. Real-time RT-PCR results indicate that Alox15 mRNA is present across all 11 different brain regions studied, with the olfactory bulb, prefrontal cortex, cerebral cortices, and the hippocampal regions displaying significant levels of expression while the hindbrain regions such as the brain stem and the spinal cord express non-significant low mRNA levels. Comparison with other lipoxygenases shows that Alox15 is the highest-expressing isoform in the rat cortex, compared to Alox5 and Alox12. Protein expression levels exhibited by Western blot were largely consistent with the results of real-time RT-PCR. The prefrontal cortex displays the highest mRNA and protein levels relative to the cerebellum, but various parts of the hindbrain and the spinal cord display almost non-quantifiable protein levels of Alox15 relative to the cerebellum in Western blot. Tissue localization via immunohistochemistry supported the results of real-time RT-PCR and Western blot. The olfactory bulb, septum, cerebral cortex, striatum, and cerebellar cortex showed moderate Alox15 staining. The distinct patterns of relative distribution and localization as elucidated by this investigation may provide insight into the roles of Alox15 in synaptic plasticity and neurodegenerative diseases. The high level of expression of Alox15 in the prefrontal cortex suggests that the enzyme may play an important role in functions such as synaptic plasticity and learning and memory. The brainstem and spinal cord showed light staining, except the inferior olivary nucleus, and some of the sensory nuclei such as the dorsal and ventral cochlear nuclei, the spinal trigeminal nucleus, and the dorsal horn of the spinal cord. In both the spinal trigeminal nucleus and the dorsal horn of the spinal cord, staining is found in the entry zone of primary afferents, i.e., the substantia gelatinosa, which is consistent with a role of Alox15 product, resolvin D1 in antinociception [38–40].

We next studied the functional role of Alox15 in prefrontal cortex, a region that showed high level of gene expression by RT-PCR, Western blot, and immunohistochemistry. In this study, we showed that Alox15 inhibition/knockdown resulted in a decrease in resolvin D1 levels. Resolvin D1 has been shown to be a potent inhibitor for inflammatory pain [38] and also blocks TNFα-induced IL-1β transcripts and regulates PMN infiltration in brain [11]. In addition, resolvin D1 has been shown to alter synaptic plasticity in the mouse spinal cord [38]. Given the potential role of RvD1 in synaptic plasticity, the effects of Alox15 inhibition/knockdown on in the prefrontal cortex were investigated using LTP, a model of synaptic plasticity underlying learning. The prefrontal cortex and hippocampus are functionally related due to projections from the CA1 region of the hippocampus to the medial and orbital prefrontal cortex [41]—forming the hippocampo-prefrontal cortex pathway, a possible pathway via which spatial information is integrated in cognitive functions and goal directed motor behavior [42, 43]. Induction of LTP in the hippocampo-prefrontal cortex pathway is characterized by an increase in the amplitude of the evoked excitatory post-synaptic field potential (fEPSP) recorded in the prefrontal cortex upon administration of a HFS to the hippocampus [44]. We observed that injection of an inhibitor or antisense oligonucleotide to Alox15 successfully prevented LTP induction in the hippocampo-prefrontal cortex pathway. The antisense knockdown of Alox15 was further corroborated by Western blot analysis showing a significant decrease in Alox15 protein expression in the prefrontal cortex after injection.

In view of the abovementioned effects of Alox15 inhibition/knockdown on LTP in the hippocampo-prefrontal cortex pathway, we postulated that Alox15 could be essential for spatial working memory, which is dependent on the pathway. Spatial working memory acts to maintain memory of the spatial information of an object, stimulus association, or location within a specific test session but not between sessions [45]. Rodents have a natural tendency to alternate their choice of goal arm in a T-maze task, which reflects an adaptation to efficiently locate resources such as food, water, and shelter in their natural environment [46] and provision of a food reward increases the motivation to perform the task [47]. Rats that received Alox15 inhibitor or Alox15 antisense oligonucleotide injection in the prefrontal cortex showed poor performance in the rewarded alternation in T-maze task compared to those injected with controls. The role of the hippocampo-prefrontal cortex pathway has been observed previously by examining performance in a delayed radial maze task, after unilateral lesion of the ventral subiculum in combination with a contralateral lesion of the prefrontal cortex. These combined lesions produced a disruption of foraging only during the test phase of this delayed win-shift task, similar to that described after bilateral inactivation of the prelimbic area [48], suggesting that transmission of information between the hippocampus and the prefrontal cortex is required when task-specific short-term memory is used to facilitate search behavior [42]. The present findings of an important role of Alox15 in prefrontal cortical function adds to our recent results that show Alox15 plays a role in supraspinal antinociception originating in the prefrontal cortex [12].

Electron microscopic analysis revealed immunolocalization of Alox15 at dendrites, which is consistent with a role in synaptic plasticity in the prefrontal cortex. NMDA receptors are important for induction of many forms of LTP including LTP in the hippocampo-prefrontal cortex pathway [49]. DHA, the major substrate of Alox15 in production of resolvin D1, has been reported to increase expression of NMDA receptor subunits important for synaptic plasticity and memory [50, 51]. Consistent with the role of PUFAs, including DHA, in promoting NMDA receptor subunit expression and inductions of LTP, dietary omega-3 deficiency reduces BDNF content and activation of NMDA receptors in the rat hippocampus [52]. In comparison, dietary enrichment with omega-3 PUFAs, including DHA, was also reported to reverse age-related decreases in the GluR2 and NR2B glutamate receptor subunits in the rat forebrain [53].

Intrathecal RvD1 has been shown to block mechanical hyperalgesia and allodynia [54, 55]. The effect of RvD1 in inhibiting neuropathic pain points to a central mechanism involving changes in neuroplasticity. RvD1 could mediate its effects on neuroplasticity through binding with known RvD1 receptors formyl-peptide receptor 2 (FPR2) and G protein-coupled receptor 32 (GPR32) [56]. Although GPR32 is a pseudogene in rats, the binding of RvD1 to GPR32 has been documented in humans to attenuate inflammation-induced mechanical hypersensitivity and spinal TNF release [55, 57]. Activation of the G protein-coupled receptors FPR2 and GPR32 by RvD1 in the central sensory pathway may alter synaptic transmission [54, 55], and one study has indicated transient receptor potential ion channel vanilloid 3 (TRPV3) as a molecular target for RvD1 [58].

In conclusion, the current findings indicate that Alox15 processing of DHA contributes to production of resolvin D1 and LTP at hippocampo-prefrontal cortex synapses and associated spatial working memory performance. Our findings point to the role of anti-inflammatory molecules such as resolvin D1 in neuroplasticity and brain signaling, and possible disturbances in this system under conditions of neuroinflammatory brain disorders and chronic neurodegeneration.

## Abbreviations

AA: Arachidonic acid
AD: Alzheimer’s disease
CNS: Central nervous system
DHA: Docosahexanoic acid
DMSO: Dimethyl sulfoxide
fEPSP: Field excitatory post-synaptic potential
HFS: High-frequency stimulation
HPC: Hippocampus
IOC: Input/output curve
iPLA_2_: Calcium independent phospholipase A_2_
LTP: Long-term potentiation
NPD1: Neuroprotectin D1
PLA_2_: Phospholipase A_2_
PUFA: Polyunsaturated fatty acid
RvD1: Resolvin D1
RvD1: Resolvin D2
SDS-PAGE: Sodium dodecyl sulfate polyacrylamide gel electrophoresis
